# Cholesterol regulates plasma membrane bending by prominin-family proteins

**DOI:** 10.1101/2023.11.08.566258

**Published:** 2023-11-10

**Authors:** Tristan A. Bell, Bridget E. Luce, Pusparanee Hakim, Hiba Dardari, Virly Y. Ananda, Tran H. Nguyen, Arezu Monshizadeh, Luke H. Chao

**Affiliations:** 1 Department of Molecular Biology, Massachusetts General Hospital, Boston, MA, 02114; 2 Department of Genetics, Blavatnik Institute, Harvard Medical School, Boston, MA, 02115

**Keywords:** prominin-1, extracellular vesicles, membrane biophysics, protein conformation, cholesterol-binding protein

## Abstract

Prominin-1 (Prom1) is a pentaspan membrane protein that associates with curved regions of the plasma membrane. Prom1 localizes to cholesterol-rich domains and requires membrane cholesterol to support membrane remodeling. Membrane bending activity is particularly evident in photoreceptors, where Prom1 mutations cause loss of outer segment disk homeostasis leading to cone-rod retinal dystrophy (CCRD). However, the mechanistic link between prominin-dependent cholesterol binding, membrane remodeling, and retinal disease remains unclear. Here, we characterize the membrane bending function and specific cholesterol binding activity of Prom1 and its proposed homolog Tweety homology 1 (Ttyh1) in extracellular vesicles (EVs). Prom1 and Ttyh1 induce formation of EVs in cultured mammalian cells that are biophysically similar. Though both proteins bend membranes and form EVs at the plasma membrane, Ttyh1 lacks a stable interaction with cholesterol that is present in Prom1. Correspondingly, Ttyh1 forms EVs that are more deformed than those produced by Prom1. An evolutionarily conserved and retinal disease-associated Prom1 residue (Trp-795) is necessary for cholesterol binding, EV membrane deformation, and efficient trafficking to the plasma membrane. Removal of *N*-glycan moieties from Prom1 biases the enzyme toward a cholesterol-bound state. We propose that Prom1 and Ttyh1 are both members of a single prominin family of membrane bending proteins, that Ttyh1 is a constitutively active member of this family, and that Prom1 is regulated by cholesterol binding and *N*-glycosylation. These findings shed light on mechanisms of prominin family function in disease and help unify models of prominin function across diverse cell types.

## Introduction

Mammalian cells interact with the extracellular environment through proteins, lipids, and glycans at the plasma membrane. Organized protrusive structures of the membrane such as microvilli and cilia are hotspots for nutrient absorption, cell cycle regulation, and extracellular signaling^1,2^. Membrane protrusions are organized both by interactions with the cytoskeleton and by sorting of proteins and lipids into membrane microdomains^2-4^. Prominin-1 (Prom1) is a five-transmembrane pass integral membrane protein that localizes specifically to cholesterol-rich membrane microdomains associated with membrane protrusion^5^. Prom1 was first characterized as the target of AC133-1, a monoclonal antibody raised against pluripotent human epithelial stem cells^6,7^. Since then, Prom1 has been identified in the apical membranes of most epithelial and neuroepithelial cell types, but is only natively recognized by AC133-1 in stem cells and photoreceptors^8-10^. In photoreceptors, Prom1 forms complexes with Protocadherin-21 (Pcdh21) that are necessary for normal membrane disc biogenesis^11-19^, and several Prom1 mutations associate with hereditary cone-rod retinal dystrophy (CRRD)^11,20-23^. Animals also express at least one paralogous prominin protein (Prominin-2) that is present in most non-retinal cell types^24^.

When overexpressed, Prom1 dramatically reorganizes the plasma membrane of cultured mammalian cells into long protrusions^25,26^. Small-molecule inhibitors of actin and tubulin do not impair the tubulation phenotype, indicating that cytoskeletal interactions are not required for membrane bending^25,26^. Accordingly, Prom1 interacts with cholesterol in cell membranes, and cells treated with cholesterol biosynthesis inhibitors or depletion agents do not exhibit membrane tubulation, suggesting that cholesterol regulates Prom1 function^25,26^.

Prom1 also induces formation of extracellular vesicles (EVs) that bleb from the apical plasma membrane of differentiating epithelial stem cells^27^. Large Prom1 EVs (500-700 nm in diameter) appear to form from bulk release of membrane from the cell midbody, whereas small Prom1 EVs (< 250 nm in diameter) likely emanate from organized membrane protrusions such as microvilli and cilia^27-29^. Prom1 EVs can be readily detected in saliva, urine, semen, neural tube fluid, and lacrimal fluid of healthy adults^28,30^.

Recently, the Tweety homology (Ttyh) protein family was hypothesized to be a distant homolog of the prominins^25^. Ttyh proteins share prominins’ five-transmembrane topology but have smaller extracellular domains^31^. Most animals have three paralogous Ttyh proteins (Ttyh1, Ttyh2, and Ttyh3)^32^ that all are predominantly expressed in neural tissues^33-38^. Notably, overexpression of Ttyh1 in cultured cells induces plasma membrane tubulation that is strikingly similar to that observed with Prom1^32,39-41^.

Understanding Prom1 membrane bending is foundational for understanding retinal disease and stem cell development, but the relationship between Prom1 and cholesterol that underlies membrane reshaping remains unclear. Here, we reconstitute and purify Prom1 EVs to characterize Prom1 function in a native membrane bilayer. Prom1 forms a stable interaction with cholesterol that is resistant to delipidation by nonionic detergent. Mutation of a residue implicated in retinal disease stabilizes cholesterol binding and negatively regulates membrane bending. *N*-glycosylation is also necessary for dynamic cholesterol binding. We further demonstrate that Prom1 homolog Ttyh1 forms EVs similar to Prom1 EVs but does not co-purify with endogenous cholesterol. We propose that cholesterol binding allosterically regulates membrane bending by prominins, and that the Ttyh proteins are prominin family members biased toward constitutive membrane bending activity. These findings contribute to a mechanistic foundation for distinct prominin function in different tissues, including the role of Prom1 in retinal homeostasis and disease.

## Results

### Reconstitution and purification of Prom1 EVs

**Figure 1.**
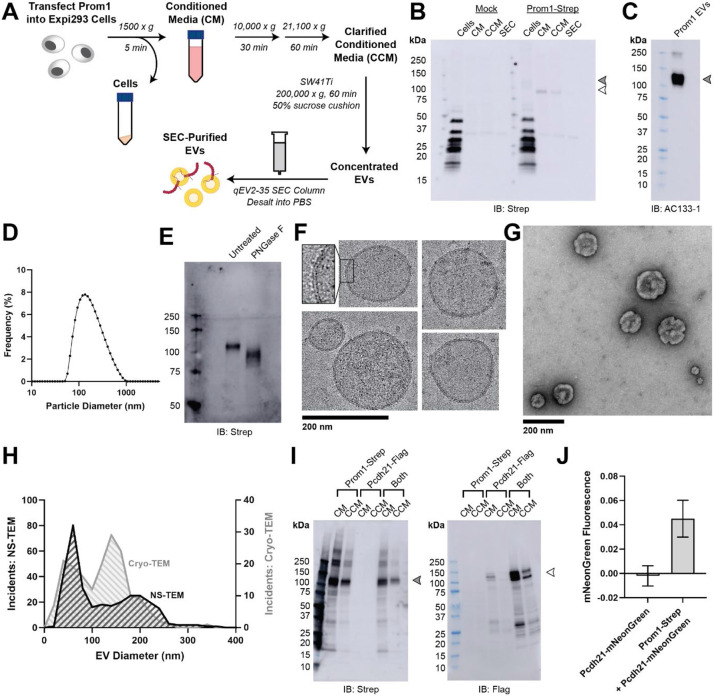
**(A)** EV expression and purification protocol. **(B)** Anti-Strep immunoblot of cell pellet, conditioned media (CM), clarified conditioned media (CCM), or SEC-purified EVs from mock-transfected or Prom1-Strep-transfected Expi293 cells. Filled and empty arrows indicate expected molecular weights of native and de-glycosylated Prom1, respectively. **(C)** AC133-1 immunoblot of SEC-purified Prom1 EVs. **(D)** Dynamic light scattering measurement of purified Prom1 EV solution size. **(E)** Anti-Strep immunoblot of Prom1 EVs treated with or without PNGase F to remove *N*-glycan moieties. **(F)** Cryo-TEM images of purified Prom1 EVs. Inset image is magnified to emphasize membrane bilayer density. Images are lowpass filtered to 5 Å to enhance contrast. **(G)** NS-TEM images of purified Prom1 EVs. **(H)** Measured diameters of Prom1 EVs from Cryo-TEM or NS-TEM images. (n = 322 and n = 176 for NS-TEM and cryo-TEM measurements, respectively.) **(I)** Anti-Strep (Prom1) and anti-Flag (Pcdh21) immunoblots of CM and CCM from cells transfected with Prom1-Strep, Pcdh21-Flag, or both. Note that Pcdh21 is only detected in CCM when co-expressed with Prom1. Filled and empty arrows indicate expected molecular weights of lycosylated Prom1-Strep and Pcdh21-Flag, respectively. **(J)** Fluorescence measurement of Pcdh21-mNeonGreen co-immunoprecipitated with Prom1-Strep.

To understand how Prom1 interacts with and reshapes native membranes, we sought a method to purify Prom1 without disturbing protein-lipid interactions. Because Prom1 is endogenously detectable in EVs^28^, we asked whether Prom1 induces EV formation in a cell culture system. C-terminally strep-tagged Prom1 was detected in immunoblots of the conditioned media from transfected cells, but not in cells treated with a no-plasmid mock transfection (Fig 1A, 1B). To validate that the ~120 kDa Strep-reactive band we observe is indeed Prom1-Strep, we immunoblotted with the Prom1-specific antibody AC133-1^42^ and observed robust staining (Fig 1C). To confirm that EV production is not an artifact of transfection, we introduced Prom1-Strep into Expi293 cells by lentiviral transduction to generate a stable polyclonal overexpression cell line. A Strep-reactive band consistent with mature Prom1 was detectable in the conditioned media from these cells over several rounds of cell passaging (Fig S1A). We assessed the protein composition of Prom1 EVs using SDS-PAGE and silver nitrate staining. Prom1 was a primary constituent of our samples alongside several other proteins (Fig S1B). We cannot conclude whether these proteins specifically interact with Prom1 in EVs, are hitchhikers enclosed within the EVs, or originate from endogenous co-purifying EVs.

We next purified the Prom1 EVs using differential centrifugation, ultracentrifugal concentration, and size-exclusion chromatography (SEC) based on isolation methods previously described for endogenous small Prom1 EVs (Fig 1A)^28,43^. Solution size measurement with dynamic light scattering (DLS) indicated that purified Prom1 EVs are monodisperse with an average diameter of 164 ± 14 nm, considerably larger than the 50-80 nm EVs measured by negative-stain transmission electron microscopy (NS-TEM) in samples purified from biological fluids^28^ (Fig 1D). Upon treatment with PNGase F, an enzyme that cleaves *N*-glycan groups from proteins, the ~120 kDa Prom1 gel band shifted down to its predicted molecular weight of 102 kDa (Fig 1E).

To verify that the purified particles are truly EVs (secreted particles with intact membrane bilayers), we vitrified purified samples and imaged them using transmission cryo-electron microscopy (cryo-TEM) (Fig 1F). We observed spherical EVs with bilayer membranes (Fig 1F, inset). The diameters of EVs measured by cryo-TEM was bimodal with an average diameter of 117 ± 58 nm, somewhat lower than our measurements from DLS (Fig 1H). We next used NS-TEM to directly compare our reconstituted Prom1 EVs with previously characterized endogenous Prom1 EVs^28^ (Fig 1G). We observed a distribution of largely circular EVs ranging in size from ~50 nm to ~250 nm in diameter, but more skewed toward smaller diameters than observed in the cryo-TEM data (Fig 1H). In addition, EVs had rough edges and internal depressions in NS-TEM, a characteristic feature of EV fixation^28^ (Fig 1H). Because solution DLS measurements (164 ± 14 nm) suggest larger EV diameters than our NS-TEM (123 ± 73 nm) or cryo-TEM (117 ± 58 nm) measurements, we speculate that sample fixation or vitrification may induce deformation and fission in the reconstituted EVs. This effect may have similarly impacted previous characterization of endogenous Prom1 EVs^28^. We conclude that reconstituted Prom1 EVs have similar morphology to endogenous EVs, but may be slightly larger in size.

We next asked whether Prom1 in purified EVs retains known functional behavior of Prom1 from endogenous membranes. Previous studies established that Prom1 stably binds Pcdh21 in photoreceptor outer segments to stabilize intermembrane tethers^11,12^. To determine if Prom1 can bind Pcdh21 in our EV samples, we co-transfected Expi293 cells with Strep-tagged Prom1 and Flag-tagged Pcdh21 and looked for the presence of each component in cells, conditioned media (CM), and clarified conditioned media (CCM). Pcdh21 was only observed in CCM samples when co-expressed with Prom1, indicating that Prom1 is necessary to traffic Pcdh21 into this class of EVs (Fig 1I). To further establish the interaction, we co-expressed Prom1-Strep with mNeonGreen-tagged Pcdh21 and immunopurified Prom1 from EVs solubilized with 1% n-dodecyl-ß-D-maltoside (DDM). Pcdh21-associated mNeonGreen robustly co-purified with detergent-solubilized Prom1 compared to a control condition lacking Prom1, indicating that the two proteins physically interact in purified EVs (Fig 1J).

### Mutations in the Prom1 transmembrane domain impair EV formation

Prom1 binds cholesterol in native membranes^26^, but it is not known how interaction with cholesterol regulates membrane bending and EV formation. We analyzed the sequence of human Prom1 to identify cholesterol recognition amino acid consensus sequences (CRAC, $\left[L∕V]\text{-}X_{1-5}\text{-}[Y∕F]\text{-}X_{1-5}\text{-}[K∕R\right]$ or mirrored CRAC (CRAC, $\left[K∕R]\text{-}X_{1-5}\text{-}[Y∕F]\text{-}X_{1-5}\text{-}[L∕V\right]$ sequences in the transmembrane domains, as these motifs often predict cholesterol binding in membrane proteins^44^. Human Prom1 contained four CRAC and two CARC motifs, of which none were completely conserved among metazoans and only one (CRAC-3) was modestly evolutionarily conserved (Figs S2A, S2B). We therefore turned to a more comprehensive evolutionary analysis of prominin proteins to identify evolutionarily conserved Prom1 transmembrane residues.

**Figure 2.**
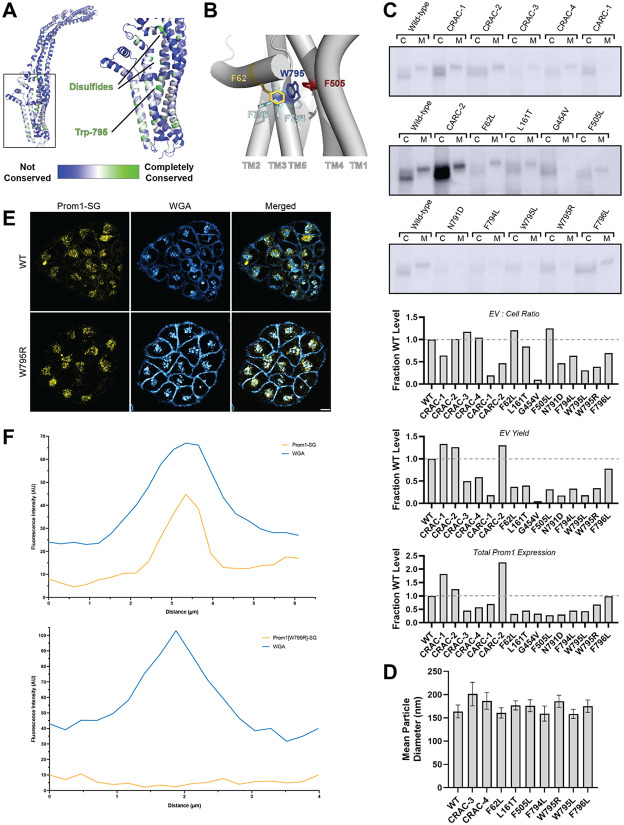
**(A)** AlphaFold2 model of human Prom1^46^ with residues color-coded by level of conservation across a representative alignment of metazoan prominin sequences. **(B)** Possible allosteric network between Trp-795 and several adjacent aromatic residues in human Prom1. **(C)** Anti-strep immunoblots of cellular and media (CCM) pools of various Prom1-Strep mutants, and quantification of those signals. **(D)** Mean particle diameter of purified Prom1-Strep EVs measured by dynamic light scattering. Error bars indicate S.D. (n = 5). **(E)** Confocal fluorescence microscopy images of HeLa cells stably expressing WT (top) or W795R (bottom) Prom1-StayGold (yellow), stained with wheat germ agglutinin (WGA) (blue). Scale bar is 10 μm. **(F)** Line scan traces across cell junctions for Prom1-StayGold and WGA signal for WT (top) and W795R (bottom) Prom1.

We curated prominin sequences from across eukaryotes, considering a prominin to be a sequence with five predicted transmembrane helices, two large extracellular loops, two small intracellular loops, and homology to annotated metazoan prominin sequences. Putative prominin sequences were identified across metazoans as well as in fungi, excavates, SAR, and green plants (Figs S3A, S3B). Strikingly, Trp-795 was nearly perfectly conserved across metazoa and eukaryotic outgroups, making it by far the most conserved residue (excluding Cys residues positioned to form disulfides in AlphaFold2 models) across the curated prominin sequences (Figs 2A, S6A). This result was of particular interest as a missense mutation at this site (W795R) is implicated in hereditary CCRD cases^45^. We selected Trp-795, Leu-161, Gly-454, and Asn-791 as residues with evolutionary evidence for potential prominin function.

We generated Prom1-Strep variants with mutations to disrupt each CRAC or CARC motif, point mutations targeting evolutionarily conserved residues, and point mutations directed against residues predicted to be in close contact with Trp-795 in Alphafold2 models (Fig 2B, Table ST1)^46^. We then assessed EV formation by quantifying Prom1-Strep signal secreted in EVs or retained in the cellular membranes. We found that mutations disrupting the CARC-1 or CARC-2 sites decreased the proportion of Prom1 secreted in EVs, as did point mutations to Gly-454 or Trp-795 and neighboring residues (Fig 2C). In most cases, this effect arose from both decreased expression of Prom1 and increased retention of protein in cells. However, the CARC-2 mutant was more highly expressed than WT, with the entire effect arising from cellular membrane retention. To better characterize the morphology of the Prom1 variants, we expressed and purified a subset of mutant Prom1 EVs at larger scale. Each of the mutants produced EVs that DLS analysis indicated to be monodisperse and 150-200 nm in diameter, similar to WT Prom1 EVs (Fig 2D). Thus, transmembrane domain mutations in Prom1 primarily alter the quantity of EVs produced rather than EV size.

To better understand the mechanism of the reduction in Prom1 EV formation, we engineered HeLa cells stably expressing C-terminally StayGold-tagged WT (Prom1-SG) or W795R Prom1 (Prom1[W795R]-SG) under the EF1a promoter. Prom1-SG signal colocalizes with Wheat Germ Agglutinin (WGA)-stained plasma membrane and intracellular vesicles, but Prom1[W795R]-SG concentrated on WGA-positive intracellular vesicles (Fig 2E). Despite the apparent low signal of plasma-membrane localized Prom1-SG, line scan analysis shows correlation between Prom1-SG signal across the cell junctions (Fig 2F top, Fig S4 left). In contrast, there is a marked absence of Prom1[W795R]-SG signal localized on the plasma membrane (Fig 2F bottom, Fig S4 right). We infer that fluorescent WT Prom1 is trafficked to the plasma membrane far more efficiently than the W795R mutant.

### Prom1 mutants adopt different cholesterol binding states

**Figure 3.**
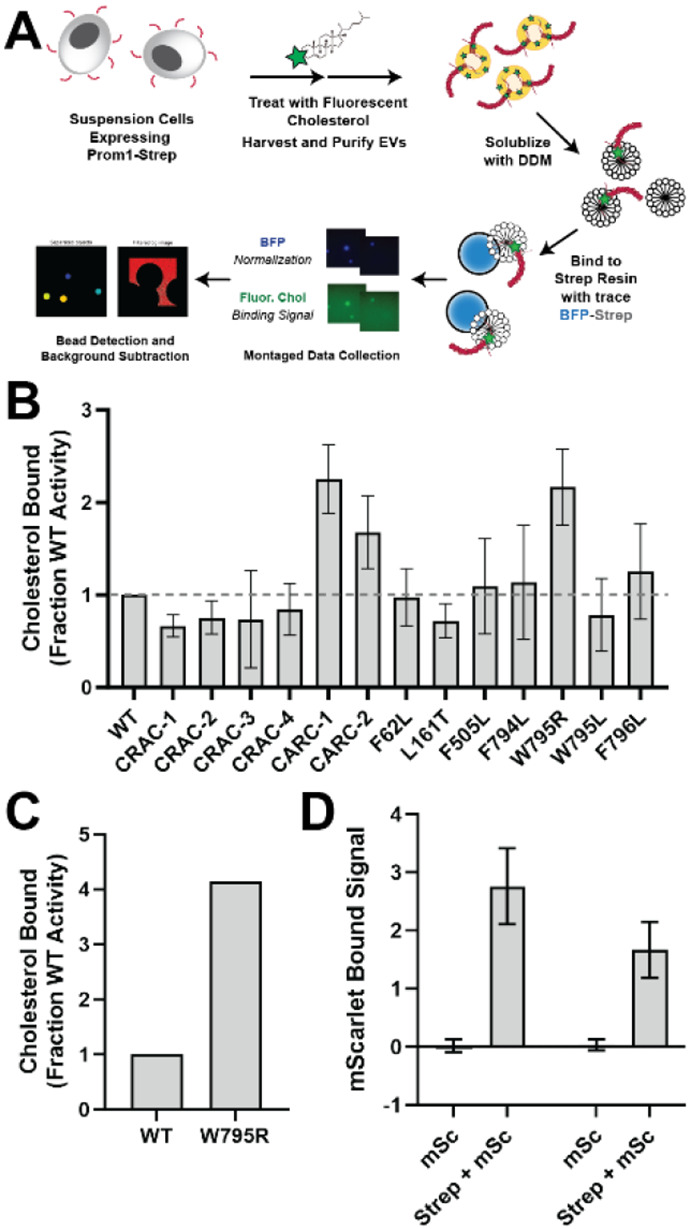
**(A)** Cholesterol co-immunoprecipitation (Chol-IP) assay graphic protocol. **(B)** BODIPY-cholesterol binding measurements for WT and mutant variants of Prom1. Error bars indicate S.D. (n = 3). **(C)** AlexaFluor647-cholesterol binding measurement for WT and W795R Prom1 **(D)** Red fluorescence signal from anti-Strep immunopurification of DDM-solubilized EVs from cells expressing Prom1-mScarlet (“mSc”) or both Prom1-mScarlet and Prom1-Strep (“Strep + mSc”). Error bars indicate S.D. (n = 3).

Prom1 localizes to cholesterol-rich microdomains of the plasma membrane.^26^ Because W795R Prom1 exhibited reduced EV formation and impaired trafficking to the plasma membrane, we hypothesized that the mutation may alter cholesterol binding. To test this hypothesis, we developed a cholesterol co-immunopurification assay (hereafter referred to as chol-IP) to quantify interaction of Prom1 with fluorophore-labeled cholesterol (Fig 3A). Prom1-Strep was transfected into Expi293 cells, and cells were labeled with a low concentration of fluorescent cholesterol. EVs were purified from the conditioned media, solubilized with nonionic detergent (DDM), and immunoprecipitated with Strep resin trace labeled with blue fluorescent protein (mTagBFP2-Strep). We then collected epifluorescence micrographs of the resin particles, computationally segmented and filtered each image, and quantified bound cholesterol using mTagBFP2 as a normalizing control (Fig 3A). This method allows for sensitive quantification of bound lipid while efficiently excluding autofluorescent and refractive artifacts.

We purified BODIPY-cholesterol-labeled EVs produced by WT and mutant forms of Prom1 and subjected equal input concentrations of Prom1 to chol-IP analysis. To our surprise, none of the mutants substantially disrupted cholesterol binding, but the patient-derived W795R mutant and the two CARC domain mutants bound cholesterol at higher levels than WT Prom1 (Fig 3B). To validate that differences in fluorescence are indeed due to different quantities of bound cholesterol, we replicated the chol-IP assay with AlexaFluor647-cholesterol for the W795R mutant and also observed an increase in cholesterol binding relative to WT protein (Fig 3C). We conclude that W795R, CARC-1, and CARC-2 Prom1 are allosterically biased toward a “cholesterol-locked” state that supports more stable cholesterol binding than the distribution of states that WT Prom1 occupies. We chose to focus our efforts on the W795R mutant as it is a naturally occurring single-residue mutation with exceptional evolutionary conservation and a clinically validated disease phenotype.

To verify that W795R Prom1 does exhibit a gross oligomerization defect, we purified EVs from cells co-transfected with Strep-tagged and mScarlet-tagged Prom1 and measured co-purification of Prom1-mScarlet on Strep resin. Prom1 W795R co-purified with ~60% as much mScarlet fluorescence as WT Prom1, indicating that W795R indeed multimerizes, albeit with reduced efficiency (Fig 3D).

### Cholesterol binding is coupled to Prom1 N-glycosylation and maturation

**Figure 4.**
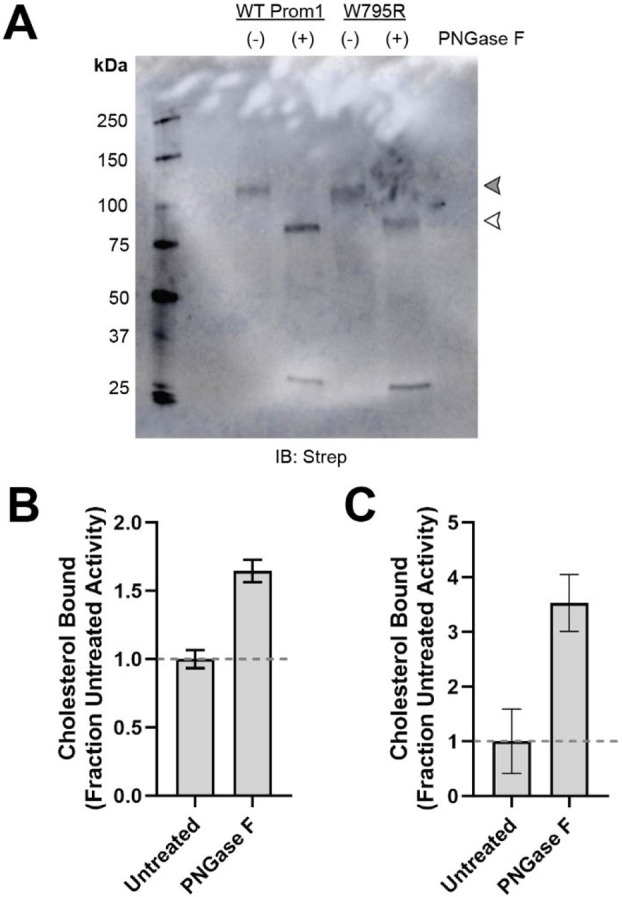
**(A)** Anti-strep immunoblot of denatured WT and W795R Prom1-Strep EVs treated with or without PNGase F to remove *N*-glycosyl moieties. Filled and empty arrowheads indicate the expected positions of fully glycosylated and deglycosylated Prom1-Strep, respectively. **(B)** Chol-IP measurement of BODIPY-cholesterol binding in Prom1-Strep EVs treated with or without PNGase F. Error bars indicate S.D. (n = 3). **(C)** Chol-IP measurement of AlexaFluor647-cholesterol binding in Prom1-Strep EVs treated with or without PNGase F. Error bars indicate S.D. (n = 3).

We consistently observed that W795R Prom1 purified in EVs ran slightly faster on SDS-PAGE gels than WT Prom1 (Fig 4A). To determine whether *N*-glycosylation alone accounts for the difference in electrophoretic mobility, we treated WT and W795R Prom1 with PNGase F. Both Prom1 variants ran on gels at the same size after *N*-glycan removal, indicating that their native size difference is due to differential glycosylation (Fig 4A). Importantly, because Prom1 EVs form by blebbing from the plasma membrane, the EV material purified from cell-free conditioned media reflects the glycosylation state of mature Prom1 from the plasma membrane rather than immature protein from the endomembrane system.

We next tested whether *N*-glycosylation alters Prom1 cholesterol binding. We purified BODIPY-cholesterol-labeled WT Prom1 EVs and incubated them with PNGase F to remove *N*-glycan moieties. Chol-IP analysis indicated that *N*-glycan removal from mature Prom1 significantly increased the level of bound BODIPY-cholesterol (Fig 4B). We similarly observed an increase in cholesterol binding for PNGase F-treated EVs labeled with AlexaFluor647-cholesterol (Fig 4C).

### Prominin homolog Ttyh1 produces EVs but does not stably bind cholesterol

**Figure 5.**
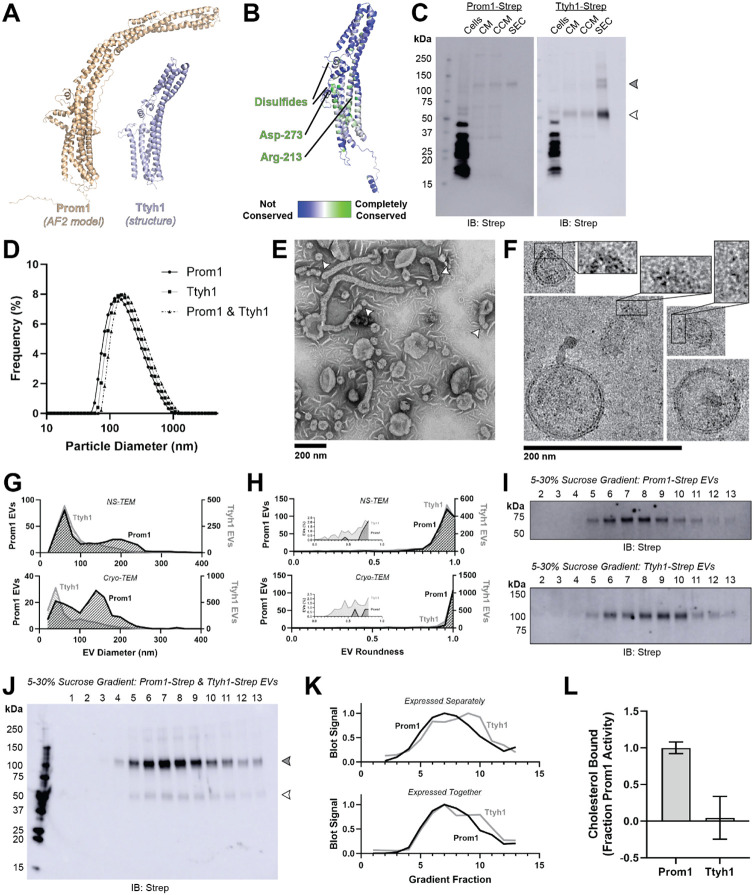
**(A)** Comparison of a Ttyh1 subunit from cryo-TEM structure 7P5J^37^ with an AlphaFold2-predicted Prom1 monomer^46^. **(B)** Residue-level conservation among metazoan Ttyh proteins plotted onto a subunit of human Ttyh1. No residue analogous to Prom1 Trp-795 is present in Ttyh. **(C)** Anti-strep immunoblot comparing Prom1-Strep EVs and Ttyh1-Strep EVs throughout different stages of purification. Filled and empty arrowheads indicate the expected positions of Prom1 and Ttyh1, respectively. Doublet and higher bands in Ttyh1 lanes are products of on-gel disulfide crosslinking in concentrated samples. **(D)** Dynamic light scattering readouts for purified Prom1-Strep, Ttyh1-Strep, or Prom1-Strep + Ttyh1-Strep co-expression EVs. **(E)** Representative NS-TEM images of Ttyh1-Strep EVs. Arrowheads indicate possible sites of EV fission. **(F)** Representative cryo-TEM images of Ttyh1-Strep EVs. Magnified insets show bilayer density at highly curved membrane segments. Images are lowpass filtered to 5 Å to enhance contrast. **(G)** Comparison of Prom1 or Ttyh1 EV diameter in NS-TEM or cryo-TEM images. (n = 322, n = 1357, n = 176, and n = 2224 for Prom1 NS-TEM, Ttyh1 NS-TEM, Prom1 cryo-TEM, and Ttyh1 cryo-TEM measurements, respectively.) **(H)** Comparison of Prom1 or Ttyh1 EV roundness in NS-TEM or cryo-TEM images. Inset plots only include EVs with roundness ≤ 0.8. (n = 322, n = 1357, n = 122, and n = 1546 for Prom1 NS-TEM, Ttyh1 NS-TEM, Prom1 cryo-TEM, and Ttyh1 cryo-TEM measurements, respectively.) **(I)** Anti-strep immunoblots of fractions from sucrose gradient equilibrium sedimentation of Prom1-Strep EVs (top) or Ttyh1-Strep EVs (bottom). **(J)** Anti-strep immunoblots of fractions from sucrose gradient equilibrium sedimentation of EVs from cells co-expressing Prom1-Strep and Ttyh1-Strep. Filled and empty arrowheads indicate the expected positions of Prom1 and Ttyh1, respectively. **(K)** Quantification of immunoblots in panels *I* (top) and *J* (bottom). **(L)** Chol-IP measurement of cholesterol binding in Prom1-Strep or Ttyh1-Strep EVs. Error bars indicate S.D. (n = 3).

Tweety homology (Ttyh) proteins are proposed prominin homologs that share the five-transmembrane topology of prominins but have shorter extracellular domains (~120 amino acids in Ttyh vs ~280 amino acids in Prom)^25^ (Fig 5A). Given the shared evolutionary history of Prom and Ttyh, we hypothesized that Ttyh may also bend membranes and produce EVs. Evolutionary analysis of metazoan Ttyh proteins does not indicate conserved CRAC or CARC sites, nor does it suggest any conserved transmembrane residue analogous to Trp-795 in metazoan prominins (Fig 5B).

We expressed Strep-tagged Ttyh1 in Expi293 cells and purified EVs using the same procedure as for Prom1 EVs (Fig 1A). We detected Ttyh1-Strep in CM, CCM, and SEC-purified EV fractions (Fig 5C). Dynamic light scattering indicated that purified Ttyh1 EVs are monodisperse with an average diameter of 180 ± 10 nm, similar to the average diameter of WT Prom1 EVs (164 ± 14 nm) (Fig 5D).

We next characterized the morphology of purified Ttyh1 EVs using NS-TEM. We found that Ttyh EVs adopted striking long and bent tubular structures with much higher frequency than Prom1 EVs (6.4% of Ttyh1 EVs versus 0.3% of Prom1 EVs, n = 1357 and n = 322 respectively) (Figs 5E, 5H). Ttyh1 EVs visualized by NS-TEM were on average smaller than Prom1 EVs, and like Prom1 EVs were smaller than expected from solution DLS measurement (Figs 5G, 5H). Furthermore, we observed that the smallest Ttyh1 EVs were similar in diameter to the short-axis caliper diameter of the tubular EVs (42 ± 6 nm) and we observed cases where tubular EVs appeared to be in the process of dividing into smaller EVs (Figs 5E, 5G). Although the NS-TEM conditions deviate from a solvated physiological state, they do indicate that Ttyh1 supports comparatively more bent membranes than Prom1.

We further analyzed Ttyh1 EVs by cryo-TEM to verify that the purified sample contained EVs. Indeed, we observed vesicles with clear bilayer membrane density (Fig 5F). Like Prom1 EVs, Ttyh1 EVs have a bimodal size distribution with local maxima around 60 nm and 140 nm (Fig 5G). We also observed a population of EVs exhibiting the tubular phenotype seen in NS-TEM that again substantially exceeded that seen with Prom1 EVs (8.8% of Ttyh1 EVs versus 0.8% of Prom1 EVs, n = 2224 and n = 176 respectively) (Fig 5H). This supports the observation that the frequency of EV bending is more frequent in Ttyh1 EVs than Prom1 EVs. We conclude that Ttyh1 supports greater membrane bending than Prom1, sufficient to induce deformation and even membrane fission under NS-TEM and cryo-TEM conditions.

To further determine similarities and differences between purified Prom1 and Ttyh1 EVs, we subjected purified EVs to equilibrium sucrose gradient sedimentation to resolve populations by density. We observed a single population of Prom1 EVs (centered on fraction 7) but resolved two distinct populations of Ttyh1 EVs with densities lower (centered on fraction 6) and higher (centered on fraction 9) than the Prom1 EVs (Fig 5I). When Prom1 and Ttyh1 were co-expressed, we observed that the resulting EVs followed the bimodal distribution of Ttyh1 EVs (Fig 5J). Immunoblots of the sucrose gradient fractions showed that co-expressed Prom1 and Ttyh1 peak in the same sucrose gradient fractions, suggesting that Prom1 and Ttyh1 co-elute in the same EV populations (Fig 5J, 5K). We conclude that Prom1 and Ttyh1 traffic to the same plasma membrane microdomains and can be secreted into the same EV membranes.

Finally, we compared stable cholesterol binding between Ttyh1 and Prom1 by purifying Ttyh1 EVs labeled with BODIPY-cholesterol or AlexaFluor647-cholesterol and subjecting them to chol-IP analysis. Surprisingly, neither cholesterol analog detectably co-purified with Ttyh1 (Figs 5L, S5). Taken together, our observations suggest that Prom1 and Ttyh1 share evolutionary history and EV formation function but differ in cholesterol interaction and the degree to which they support EV membrane bending.

### Prom1 W795R EVs are morphologically similar to WT Prom1 EVs

**Figure 6.**
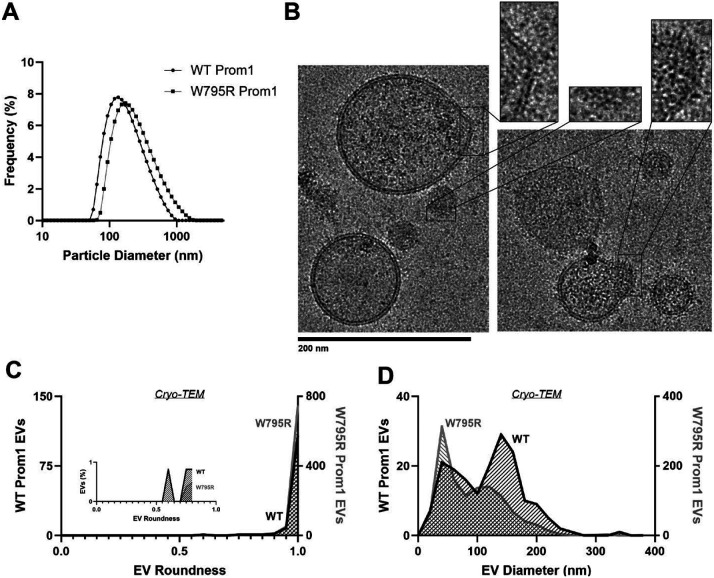
**(A)** Dynamic light scattering readouts for purified WT or W795R Prom1-Strep EVs. **(B)** Representative cryo-TEM images of W795R Prom1-Strep EVs. Magnified insets show bilayer density. Images are lowpass filtered to 5 Å to enhance contrast. **(C)** Comparison of WT or W795R EV roundness in cryo-TEM images. Inset plots only include EVs with roundness ≤ 0.8. (n= 176 and n = 1211 for WT and W795R Prom1 cryo-TEM measurements, respectively.) **(D)** Comparison of WT or W795R Prom1 EV diameter in cryo-TEM images. (n= 122 and n = 821 for WT and W795R Prom1 cryo-TEM measurements, respectively.)

Because Ttyh1 stabilizes greater membrane curvature than Prom1 but does not stably bind cholesterol, we hypothesized that cholesterol binding may negatively regulate membrane bending by prominin-family proteins. This model predicts that W795R Prom1, which binds cholesterol more stably than WT protein, should not produce EVs that exhibit the tubular morphology observed in Ttyh1 EVs. When measured by DLS, we observed a similar solution size for WT (164 ± 14 nm) and W795R EVs (186 ± 13 nm) (Figs 2D, 6A). We measured the size and shape of W795R EVs by cryo-TEM and observed largely spherical vesicles with some local deformations, similar to what we observed with WT Prom1 EVs (Fig 6B). Generally, WT and W795R Prom1 EVs have similar roundness profiles, with W795R having no vesicles that fall into our tubular morphology classification (n = 1211) (Fig 6C). Though both large and small diameter EV populations were observed by cryo-TEM, W795R Prom1 had a larger fraction of small EVs than WT Prom1, indicating possible decreased stability or increased fissile propensity in the W795R Prom1 EVs (Fig 6D).

### Cholesterol binding modulates membrane bending by Prom1

**Figure 7.**
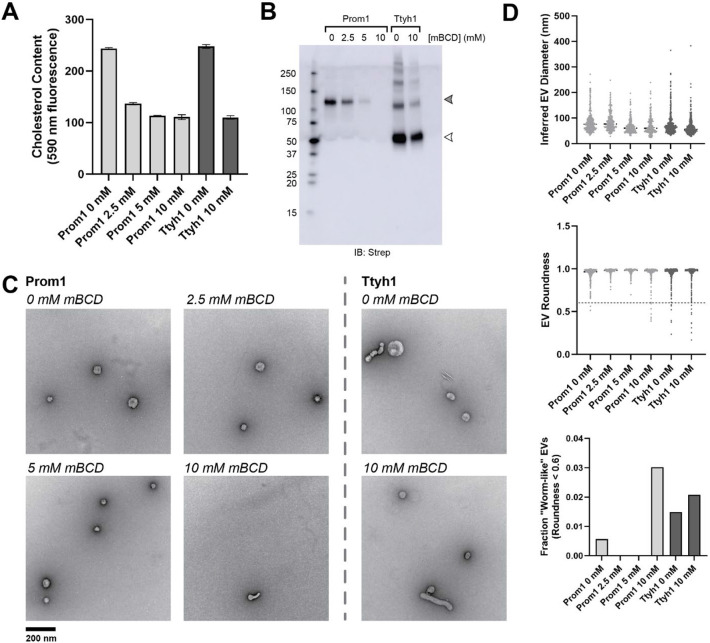
**(A)** Cholesterol content of Prom1 or Ttyh1 purified EV samples after treatment with mBCD. Error bars indicate S.D. (n = 3). **(B)** Anti-strep immunoblot of mBCD-treated and purified Prom1 or Ttyh1 EVs. Filled and empty arrowheads indicate the expected positions of Prom1 and Ttyh1, respectively. Higher-MW bands are products of on-gel disulfide crosslinking. **(C)** Representative NS-TEM images of mBCD-treated and purified EVs. **(D)** Quantification of EV diameter (*top*) and roundness (*middle*) from NS-TEM images, as well as quantification of EVs with roundness < 0.6 (*bottom*).

A further prediction of the model that cholesterol binding negatively regulates membrane bending is that depleting cholesterol from EV membranes could induce a Ttyh1-like morphology in Prom1 EVs. We tested this model directly by purifying WT Prom1 or Ttyh1 EVs and treating them with methyl beta cyclodextrin (mBCD), a compound that extracts cholesterol from membranes^26,47^. After re-purifying EV samples away from free mBCD and mBCD-cholesterol complexes, Prom1 and Ttyh1 EVs treated with mBCD (2.5-10 mM) exhibited a decrease in cholesterol content (Fig 7A). At increasing concentrations of mBCD, we observed depletion of Prom1 and Ttyh1 after EV re-purification, suggesting that cholesterol depletion may destabilize EVs (Fig 7B). Treated and untreated EVs were then analyzed by NS-TEM to compare EV size and morphology (Fig 7C). We observed a 5.4-fold increase in the fraction of Prom1 EVs that deviated from spherical membrane topology after 10 mM mBCD treatment compared to untreated EVs (3.0% versus 0.6% of EVs, n = 232 and n = 356 respectively) (Fig 7D). In contrast, Ttyh1 EVs treated with mBCD under identical conditions only showed a 1.4-fold increase in deformed EVs (2.1% versus 1.5% of EVs, n = 433 and n = 535 respectively) (Fig 7D). This indicates that EV membrane deformation is specific to Prom1 EVs rather than a nonspecific effect triggered by EV cholesterol depletion. Thus, cholesterol-depleted Prom1 mimics the membrane bending activity of Ttyh1.

## Discussion

### Prominin-family proteins Prom1 and Ttyh1 bend membranes and form EVs

Prominin and Tweety homology proteins are both biologically implicated in membrane bending processes. Prominins localize to cholesterol-rich microdomains of the plasma membrane and drive protrusion-shed EV formation in differentiating stem cells^26-28^. Tweety homology proteins are associated with dendritic spikes in healthy neurons and with tumor microtube formation in aggressive astrocytoma^32,39-41^. We report here that both Prom1 and Ttyh1 can induce EV secretion from cultured cells upon overexpression (Fig 5C). Though we do not claim that Ttyh1 endogenously induces EV formation, we note that it is sufficient to do so in a recombinant system with similar efficiency to Prom1. Given similar membrane remodeling tubulation behavior of Prom1 and Ttyh1 in cell culture^25,40^, Ttyh1 EVs may form by a similar mechanism to Prom1 EV formation.

We find that Prom1 and Ttyh1 form EVs that are of similar size, and that the two proteins exist in the same pool of EVs when co-expressed in cell culture (Figs 5J, 5K). Because Prom1 EVs arise directly from blebbing of the plasma membrane, this finding suggests that Prom1 and Ttyh1 traffic endogenously to the same lipid microdomains. Given their similarities in localization, membrane bending behavior, and transmembrane topology, we propose that Prominins and Tweety homology proteins be considered members of a broader prominin family of membrane remodeling proteins.

Membrane bending by Prom1 is critical for maintaining homeostasis in photoreceptor outer segments. Intriguingly, photoreceptors with impaired expression of the disc rim stabilizing protein Peripherin accumulate EVs 200-280 nm in diameter at the outer segment base^48^. Intriguingly, we observe formation of Prom1 EVs of relatively similar size in our reconstituted system (Fig 1D). This may be an example of endogenous regulation of Prom1-induced membrane curvature, with curvature-stabilizing proteins like peripherin preventing the evaginating membrane from budding into EVs.

### Cholesterol binding regulates membrane bending by Prom1, but not Ttyh1

We find here that Prom1 in EVs forms a stable interaction with cholesterol that is resistant to delipidation by nonionic detergent (Fig 3B). By contrast, Ttyh1 does not stably bind cholesterol (Fig 5L). Though both proteins induce release of similarly sized EVs, Prom1 EVs appear predominantly spherical when visualized by NS-TEM whereas a substantial fraction of Ttyh1 EVs appear tubular and rearrange into smaller EVs (Figs 1G, 5E). Prom1 EVs mimic the Ttyh1-like deformed morphology when cholesterol is depleted from EV membranes, indicating that cholesterol interaction negatively regulates membrane bending by Prom1 (Figs 7C, 7D).

**Figure 8.**
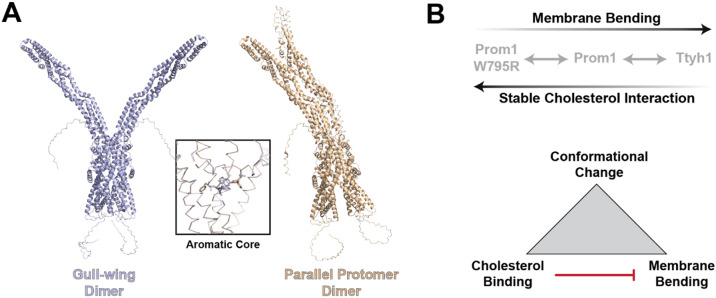
**(A)** Comparison of highest-ranked AlphaFold2 dimer models of Prom1^46^. Inset shows an alignment of the core aromatic cluster (Phe-62, Phe-505, Phe-794, Trp-795, Phe-796) between the two models indicating the highly consistent transmembrane domain prediction. **(B)** Model for coupled conformational change, cholesterol interaction, and membrane bending in prominin-family proteins.

A disease-associated mutation in a strikingly conserved residue in the fifth transmembrane helix of Prom1, W795R, substantially stabilizes cholesterol binding (Figs 2A, 3B). In AlphaFold2 models, Trp-795 forms a series of base-stacking interactions with Phe residues in neighboring transmembrane helices (Fig 2B)^46^. We find that conservative mutations in several of these adjacent aromatic residues impair EV formation by Prom1, but do not mimic the stable cholesterol binding of W795R (Figs 2C, 3B). In a comparison of the two lowest-energy AlphaFold2 dimer models of Prom1, the conformation of this aromatic core remains constant despite a global change in Prom1 topology (Figs 8A, S6B). This suggests that neither predicted Prom1 dimer reveals a potential cholesterol-locked conformation adopted by W795R Prom1. Cholesterol is asymmetrically distributed in eukaryotic membranes, with a bias toward the outer leaflet of the plasma membrane^49^. Prom1 Trp-795 is also predicted to be positioned toward the outer leaflet, possibly potentiating its cholesterol interactions. We also observe increases in cholesterol binding stability for mutations to two CARC domains in Prom1 (Fig 3B). This effect could arise because these residues interface with cholesterol or destabilize the transmembrane domain in a manner that mimics the effect of the W795R mutation. Given the poor evolutionary conservation of the two CARC motifs in human Prom1, we favor the latter explanation.

We further show that the W795R mutation prevents efficient trafficking and plasma membrane insertion of Prom1 from the endomembrane to the plasma membrane (Figs 2E, 2F). This observation explains the very low yield of W795R EVs produced in cell culture. W795R Prom1 was well expressed but strongly localized to WGA^+^ intracellular vesicles. These vesicles are believed to traffic downstream of the trans-Golgi, suggesting that the roadblock in W795R Prom1 endomembrane trafficking occurs between the trans-Golgi and the plasma membrane^50^. Dynamic cholesterol interaction and conformational changes may be required for proper maturation and trafficking of Prom1 within the endomembrane system. The altered *N*-glycan profile of W795R may also contribute to this effect, as ablation of genes involved in *N*-glycan biosynthesis inhibits Prom1 trafficking to the plasma membrane^51^ (Fig 4A).

### N-glycosylation is necessary for Prom1 function

Many membrane proteins are *N*-glycosylated in the endoplasmic reticulum, and *N*-glycans are further modified in the Golgi^52^. Prom1 is no exception, with eight predicted *N*-glycosylation sites in the large extracellular loops^7,51^. Each site is individually dispensable for Prom1 trafficking to the plasma membrane, but complete ablation of glycosylation disrupts trafficking^51^. We find that the *N*-glycan moieties on Prom1 are not only required for maturation and trafficking, but also destabilize Prom1-cholesterol interaction (Fig 4B). A previous study has reported that mature Prom1 can exist in complex or high-mannose glycosylation profiles that have distinct interaction profiles with other proteins at the plasma membrane^10^. Though our system cannot directly dissect the relationship between different *N*-glycosylation profiles and Prom1 conformation, we do note that mutations in Trp-795 appear to bias Prom1 away from complex glycosylation and toward a lower molecular weight product consistent with a high-mannose profile (Fig 4A). It is therefore possible that regulated *N*-glycosylation functionalizes Prom1 for different roles within different tissues by altering interaction with cholesterol and the degree of membrane bending that the protein supports.

### Prom1 cholesterol binding regulates membrane bending

We propose here that Prom1 forms dynamic interactions with cholesterol that contribute to regulation of membrane bending (Fig 8B). These cholesterol-dependent interactions allosterically modulate Prom1 and are functionally linked to the glycosylation state of the protein. It is important to note that the cholesterol function at the plasma membrane is highly pleiotropic. Cholesterol contributes to membrane remodeling specifically through allosteric modulation of prominins and nonspecifically through lipid microdomain organization and modulation of membrane fluidity.

The Prom1 homolog Ttyh1 lacks stable interaction with cholesterol, has no conserved analog of the Trp-795 residue, and supports greater membrane deformation than WT Prom1. Though Ttyh proteins are evolutionarily divergent from Prom proteins and the explanations for their different membrane bending behavior are likely multifaceted, we find the ability of Prom1 to mimic Ttyh1 activity upon cholesterol depletion notable. Prominins may therefore be regulated membrane benders that can fulfill different roles in different tissue types, whereas Ttyh proteins are constitutive membrane benders exclusively expressed in neuronal niches that require this function. Further study of regulated prominin-family function in different tissue contexts will be necessary to fully understand the role of prominin-dependent membrane remodeling in cellular function.

## Materials and Methods

### Expression Constructs

All Prom1 expression constructs were generated by site-directed mutagenesis from pCS2-Prom1-YFP (a generous gift from N. Sasai, Nara Institute of Science and Technology). The base Prom1 construct used is the human S1 isoform (NCBI accession NP_001139319.1)^53^. Prom1-Strep was subcloned into pLV-EF1a vector (a generous gift from K. Hochedlinger, Massachusetts General Hospital) for lentiviral transduction. Human Pcdh21 isoform 1 (NCBI accession NP_149091.1) was synthesized (GenScript) and cloned into a pCDNA3.1 vector (ThermoFisher) for mammalian cell transfection. mTagBFP2 was subcloned from pBAD (Addgene #54572, a gift from Michael Davidson) into a pET28a vector with a C-terminal Strep tag for bacterial overexpression. Ttyh1 was expressed from a pLX304 vector after addition of C-terminal Strep and His tags to an existing construct (Addgene #161676, a gift from Mike McManus). To stably express fluorescently tagged Prom1 WT and W795R variants in HeLa cells for live cell imaging, Prom1-StayGold and Prom1[W795R]-StayGold sequences were synthesized (GenScript) and cloned into P2555 vector (kind gift by S. Jakobs, Max Planck Institute for Biophysical Chemistry) to yield constructs pAH18 and pAH20, respectively.

### Cell Line Construction

Cell lines were regularly tested for mycoplasma contamination. Lentiviral transduction of Prom1-Strep into Expi293 cells was performed using a modification of published protocols^54,55^. Briefly, lentiviral particles were produced in 293T cells (ATCC) by transient transfection of pLV-EF1a-Prom1-Strep with VSVG and Delta 8.9 plasmids (a generous gift from K. Hochedlinger, Massachusetts General Hospital) using the Lipofectamine 3000 system (Thermo Fisher) and incubated overnight at 37 °C, 5% CO_2_. Expi293 cells (Thermo Fisher) raised for several passages in suspension culture were seeded onto adherent tissue culture plates in adherent culture media (DMEM (Gibco) supplemented with 10% Fetal Bovine Serum (Gibco) and 1x Penicillin/Streptomycin (Gibco)) and incubated overnight at 37 °C, 5% CO_2_ to form an adherent monolayer. Transfected cells were exchanged into fresh adherent culture media. Culture media was harvested after an additional 48 h, filtered through a 0.45 μm vacuum unit, and concentrated from 70 mL to 0.3 mL in PBS buffer by ultracentrifugation according to established protocols^55^. Adherent Expi293 cells were infected with concentrated lentiviral particles at 75% confluency in adherent culture media supplemented with 8 μg/mL polybrene (Sigma-Aldrich), then incubated for 48 h at 37 °C, 5% CO_2_. Cells were gently washed with PBS, exchanged into adherent culture media, and incubated for 24 h at 37 °C, 5% CO_2_. Subsequently, the cells were subjected to antibiotic selection by exchange into adherent culture media containing 2 μg/mL Blasticidin (Gibco) for 10 days with regular exchange into fresh selective media and passaging to prevent cells from achieving full confluency. Selection was considered complete when the majority of cells died and antibiotic-resistant foci recolonized the culture plate. The cells were then trypsinized (Gibco) and transitioned back to suspension culture in modified suspension culture media (Expi293 media (Gibco) supplemented with 1% Fetal Bovine Serum) at a density of 1.0 x 10^6^ live cells per mL of culture and incubated for 48 h at 37 °C, 8% CO_2_ with 125 rpm rotation. Once the suspension culture reached a density of 3.0 x 10^6^ live cells per mL of culture, cells were re-passaged in 1.0 x 10^6^ live cells per mL of culture in fresh modified suspension culture media supplemented with 1.5 μg/mL Blasticidin, and subsequently re-passaged into this media condition every 2 days.

For generation of stable HeLa cells expressing WT Prom1-StayGold and Prom1[W795R]-StayGold off the AAVS1 site, the donor plasmids pAH18 or pAH20 were co-transfected with the nuclease plasmid PX458-AAVS1 (kind gift by S. Jakobs, Max Planck Institute for Biophysical Chemistry) using Lipofectamine 3000 (Thermo Fisher Scientific). Transfected cells were selected with 10 μg/mL blasticidin (Gibco) starting 48 h post-transfection for 7 days. Stable clones were expanded for 10 days, and single-cell GRP-positive clones were obtained using a FACS AriaII Cell Sorter (BD Biosciences). After clonal expansion, positive clones were detected and verified by fluorescence imaging.

### Synthesis of fluorescent cholesterol analogs

BODIPY-cholesterol was procured commercially (TopFluor cholesterol, Avanti Polar Lipids) and resuspended at 1 mM in ethanol. AlexaFluor647-cholesterol was synthesized from Alkyne Cholesterol and AZDye 647 Azide Plus (Click Chemistry Tools). Briefly, 1 mM AZDye 647 Azide Plus and 2 mM alkyne cholesterol (each from a 10 mM stock prepared in anhydrous DMSO) were combined with 2 mM tetrakis(acetonitrile)copper(I) tetrafluoroborate (from a 40 mM stock prepared in ethanol) in a 350 μL reaction brought up to volume with ethanol. The reaction was incubated at 42 °C for 30 min then at 70 °C for 2 h with the reaction vessel open to allow solvent to evaporate. Cholesterol was extracted from the final mixture by diluting the solution to 650 μL with PBS buffer and adding 1 mL methanol and 0.5 mL chloroform. The mixture was centrifuged for 2 min at l4,000 x *g* and the supernatant transferred to a clean vessel, after which 1 mL of chloroform and 2 mL of glacial acetic acid were added and mixed by vortexing. The solution was then concentrated by evaporation in a SPD1010 SpeedVac instrument (Savant) until dried, then resuspended in 100 μL ethanol. This preparation was considered to be at 5 mM labeled cholesterol (assuming 100% yield) for downstream calculations.

### Prom1 and Ttyh1 EV Reconstitution

Prom1 and Ttyh1 EVs were reconstituted by expression in Expi293 suspension cells. Briefly, Expi293 cells grown in serum-free Expi293 media at 37 °C, 8% CO_2_ with 125 rpm rotation to a density of 3.0 x 10^6^ live cells per mL of culture were transiently transfected with an appropriate plasmid at 1 μg of DNA per 1 mL of culture using the Expifectamine transfection kit (Thermo Fisher) according to the manufacturer protocol. After 48 h, cultures were centrifuged for 5 min at 500 x *g*, the media discarded, and the cells resuspended in the same volume of fresh Expi293 media and returned to incubate for an additional 48 h. After this final incubation, cultures were centrifuged for 5 min at 1500 x *g* and the conditioned media transferred to clean 50 mL conical tubes.

EVs labeled with fluorescent cholesterol analogs were generated as described above with the following modifications. Two days after transfection, cells were transferred to 50 mL conical tubes, centrifuged for 5 min at 500 x *g*, then resuspended in an equal volume of Expi293 media with fluorescent cholesterol added to a final concentration of 1 μM. Cells were transferred back to shaker flasks and incubated for 24 h before conditioned media was harvested.

### EV Purification

Conditioned media was clarified immediately after harvest by centrifuging for 30 min, 10,000 x *g* at 4 °C; then transferring the supernatant to clean tubes and centrifuging again for 1 h, 21,100 x *g* at 4 °C. The supernatant was transferred to clean 50 mL conical tubes and stored at 4 °C until ready for further purification. Clarified conditioned media was transferred to Seton 7030 tubes and each tube underlaid with a 100 μL cushion of 50% sucrose. Tubes were centrifuged in an SW-41 Ti rotor (Beckman Coulter) for 1 h, 36,000 rpm at 4 °C, then ≥ 200 μL of volume was harvested from the bottom of each tube. In cases where the total harvested volume exceeded 500 μL, the harvested volume was diluted to 11 mL with sterile-filtered PBS buffer, transferred to a final Seton 7030 tube, underlaid with a 100 μL cushion of 50% sucrose, re-centrifuged as described above, and 500 μL of volume harvested from the bottom of the tube. Concentrated EVs were then purified by size exclusion chromatography into sterile-filtered PBS buffer using qEV2-35 nm gravity columns (Izon) at ambient temperature (0.5 mL load volume, 2.5 mL void volume, 1.2 mL harvest volume). Purified EVs were stored at 4 °C for up to 8 weeks, over which time no evidence of Prom1 degradation was observed.

### Immunoblots

Protein samples were run on 4-20% or 7.5% Mini-PROTEAN TGX SDS-PAGE gels (BioRad), then transferred to PVDF membranes using the TurboBlot semi-dry transfer system (BioRad). Blots were washed briefly 3 times with 10 mL of PBST buffer, then incubated for 1-2 h at room temperature in PBST with blocking agent (5 mg/mL Bovine Serum Albumin (Sigma Aldrich) for anti-Strep blots, 5% nonfat dry milk for all other blots). Blocking solution was removed and primary antibody solution in PBST with blocking agent (1:2000 rabbit anti-Strep (Abcam #76949), 1:4000 mouse anti-Flag (Millipore Sigma #F3165), 1:2500 mouse AC133-1 (Miltenyi #130-111-756)) for 2 h at ambient temperature or for 12-72 h at 4 °C. The blots were then washed 3 times for 5-10 min with 10 mL PBST. Secondary antibody solution (1:5000 ECL anti-Mouse (Cytiva) or 1:10,000 ECL anti-Rabbit (Cytiva)) in PBST with appropriate blocking agent was then added to the blots and incubated for 1 h at ambient temperature. Blots were incubated with 5 mL Western Lighting ECL solution (PerkinElmer) for 1 min and imaged using the chemiluminescence setting on an Amersham 680 gel imager (GE Healthcare). Blots were adjusted for brightness and contrast using GIMP (GNU Project) and annotated with Illustrator (Adobe).

### Immunoblot Quantification

Samples were treated first with 250 U PNGase F (New England Biolabs) for 2 hr at 37 °C to condense each protein to a single molecular weight, then with 4 mM N-propyl maleimide (Millipore Sigma) for 1-2 h at 37 °C to block reactive cysteines and prevent on-gel disulfide crosslinking. Reactions were quenched by addition of reducing SDS-PAGE loading buffer. After blotting and imaging, images were adjusted for brightness and contrast and subjected to digital densitometry with ImageJ^56^. Resulting measurements were reported normalized to WT Prom1 on each blot to allow comparisons between blots.

### Dynamic Light Scattering

Dynamic light scattering (DLS) measurements were performed using an SZ-100 Nano Particle Analyzer (Horiba). EVs diluted in PBS to a volume of 1 mL were transferred to a disposable plastic cuvette (Fisher) and measurements were taken using settings for polydisperse liposomes in aqueous buffer. All measurements were taken at 25 °C in multiple technical replicates to control for instrument sampling error.

### Glycosylation Assays

PNGase F (New England Biolabs) was used to remove *N*-glycan moieties from proteins. For *N*-glycan removal under denaturing conditions, the PNGase F kit was used as directed. For *N*-glycan removal under native conditions, 250 U of PNGase F enzyme were added to EVs in PBS buffer in a 25 μL reaction, then incubated for 2 h at 37 °C.

### Silver stain

EV samples were run on 7.5% SDS-PAGE Tris-Glycine gels and stained with Pierce Silver Stain for Mass Spectrometry (Thermo Scientific) according to the manufacturer’s protocol.

### Cryo-TEM Sample Preparation and Imaging

Prom1 and Ttyh1 EVs were vitrified on 300-mesh gold Quantifoil R 1.2/1.3 + 2 nm Carbon grids (Electron Microscopy Sciences). Briefly, grids were glow discharged in an EasiGlow device (Pelco) set to 5 mA, 30 s, 0.39 mbar, with a 15 s vacuum hold time. The grids were then treated with 5 μL of purified EVs, incubated for 60 s to allow EVs to adhere to the carbon layer, then blotted with a VitroBot Mark IV (Thermo Scientific) set to 22 °C, 5 s blot time, +15 blot force, 100% humidity; and plunge frozen in liquid ethane. Vitrified samples were imaged on a Titan Krios microscope (Thermo Scientific) with a Falcon 4 direct electron detector (Thermo Scientific) using counted detection mode, 105,000 x nominal magnification, 0.83 Å pixel size, with 49-frame fractionated collection, 49.8 e^−^/Å^2^ total dose, and defocus ranging from −0.8 to −2.0 μm in 0.1 μm increments. Images were processed and analyzed with CryoSparc v. 4.2.1 (Stuctura Biotechnology). Vesicles were defined and diameter (all EVs) and roundness (only EVs completely visible on one micrograph) were calculated using custom scripts that extend CryoSparc, made publicly available at https://github.com/tribell4310/vesicle-quantification.

### NS-TEM Sample Preparation and Imaging

Formvar carbon film 400-mesh copper grids (Electron Microscopy Sciences) were glow discharged in an EasiGlow device (Pelco) set to 30 mA, 30 s, 0.39 mbar, with a 15 s vacuum hold time. 5 μL of EV sample was applied to a glow-discharged grid and incubated for 60 s at room temperature. The grid was then blotted manually with filter paper (Whatman #43), briefly washed 3 times with 20 μL of PBS buffer, blotted, washed 2 times with deionized water, blotted, washed 1 time with 1.25% (w/v) uranyl formate (Electron Microscopy Sciences), and blotted. The grid was then floated for 10 s on a 20 μL of drop of 1.25% uranyl formate, blotted, and allowed to air dry. Imaging was performed on a Tecnai T12 transmission electron microscope (FEI) equipped with an XR16 detector (AMT) operated at an accelerating voltage of 120 kV, 30,000 x nominal magnification, 4.32 Å pixel size, and −1.5 μm defocus. Pixel size was manually calibrated prior to image acquisition using a dedicated calibration waffle grid (Ted Pella). Vesicles were defined and analyzed as described in CryoSparc (Structura Biotechnology) as described above for cryo-TEM data.

### Evolutionary Analysis of Prom and Ttyh proteins

We identified sequences homologous to human Prom1 using BLAST^57^ and InterPro^58^. Putative prominin sequences were curated to include only sequences with five predicted transmembrane helices by TMHMM^59^ in a 2+2+1 pattern, containing large extracellular loops (> 300 amino acids) and 2 small intracellular loops (< 25 amino acids). This broad search revealed prominin sequences across the eukaryotic tree ranging from metazoans to green plants. Multiple sequence alignment was performed using MAFFT^60^ and phylogenetic relationships inferred using IQ-TREE with MODELFIND for evolutionary model selection^61,62^. Branch supports were calculated using the approximate likelihood ratio test^63^. A smaller tree was also constructed spanning only metazoan sequences, with fungi included as an outgroup for rooting. For direct comparison between Prom and Ttyh sequences, homologs of human Ttyh1 were identified for all species included in the prominin metazoan tree and aligned as described above. Figures showing multiple sequence alignments were generated using JalView^64^. Figures showing trees were generated using IcyTree^65^.

Conservation of each residue within the Prom and Ttyh metazoan trees was calculated using the Livingstone and Barton algorithm implemented in JalView^66^. Conservation scores were then plotted onto the AlphaFold2 structure model^46^ of human Prom1 or a subunit of the solved cryo-EM structure of human Ttyh1^37^ using PyMol (Schrödinger).

### Live Cell Fluorescence Microscopy and Analysis

Confluent monoclonal HeLa cells stably expressing WT Prom1-StayGold and Prom1[W795R]-StayGold were harvested, seeded onto 35 mm glass-bottom dishes (MatTek Life Sciences) coated with poly-D-lysine (0.1 mg/mL) and allowed to grow overnight at 37°C under 5% CO_2_. Cells were stained with wheat germ agglutinin (WGA) conjugated with Alexa Fluor 647 (W32466, Thermo Fisher Scientific) at 5 μg/mL for 10 min at 37 °C, then washed twice with 1x PBS. Cells were placed in Live Cell Imaging Solution (Invitrogen) prior to imaging using Nikon A1R HD25 point scanning confocal with GaAsP and PMT detectors, equipped with an Apo TIRF 60x/1.49 NA objective lens and Ti2 Z-drive. Temperature, humidity, and CO_2_ concentrations were controlled with a Live Cell environmental chamber (Oko-Lab). Image acquisition were done using NIS-Elements (Nikon Instruments Inc.) and subsequent analysis were performed using Fiji^67^.

### Protein Expression and Purification

A plasmid encoding mTagBFP2-Strep was transformed into *E. coli* BL21 (DE3) pLysS, grown at 37 °C in LB media to OD_600_ 0.5-0.7, induced with 0.5 mM IPTG (Gold Biotechnology), and harvested after 3 h of expression. Cells were resuspended in 25 mL per liter of culture of Buffer A (25 mM HEPES-NaOH pH 7.5, 500 mM NaCl, 20 mM imidazole, 0.5 mM dithiothreitol (DTT)) supplemented with 10 μM leupeptin (Sigma Aldrich), 1 μM pepstatin A (Sigma Aldrich), 1 mM phenylmethylsulfonyl fluoride (Sigma Aldrich), 1 mg/mL chicken egg lysozyme (Fisher), and 250 U benzonase nuclease (Sigma Aldrich); and incubated with stirring for 1 h at 4 °C. Cells were then sonicated for 3 min in an ice/water bath with 5 s on / 10 s off pulses. The lysate was then clarified by centrifugation in a JA-25.5 rotor (Beckman Coulter) for 45 min, 15,000 rpm, 4 °C. The supernatant was then loaded onto two 5-mL HisTrap columns (Cytiva) plumbed in series equilibrated in Buffer A using a peristaltic pump at 1.5 mL / min flow rate. The column was washed with 100 mL of Buffer A and eluted with 20 mL of Buffer B (25 mM HEPES-NaOH pH 7.5, 500 mM NaCl, 300 mM Imidazole, 0.5 mM DTT). The protein was found to be ~95% pure by SDS-PAGE, and the concentration of the eluted material was measured using absorbance signal at 280 nm. The samples were divided into small aliquots, flash frozen in liquid nitrogen and stored at −80 °C.

### Chol-IP Assays

Prior to running chol-IP assays, input EVs were quantified by SDS-PAGE and immunoblotting to ensure equal inputs of Prom1-Strep and/or Ttyh1-Strep in each assay. EVs were mixed 4:1 with buffer CIA (25 mM HEPES-NaOH pH 7.8, 150 mM NaCl, 5 mM CaCl_2_, 5% n-dodecyl-beta-maltoside (DDM) (Anatrace)) and incubated for 1 h at 4C with end-over-end rotation, protected from ambient light. During incubation, 0.025 μL of StrepTactinXT 4Flow resin (IBA) per condition was equilibrated with buffer CIB (25 mM HEPES-NaOH pH 7.8, 150 mM NaCl, 5 mM CaCl_2_, 1% DDM) in a single pooled reaction. mTagBFP2-Strep was added to the resin at a ratio of 2 μL of 10 nM mTagBFP2-Strep per 1 μL of resin, and incubated for 15 min at 4 °C with end-over-end rotation, protected from ambient light. After one additional wash with buffer CIB, the resin was then divided equally across low-binding 1.5 mL tubes (USA Scientific) so that each condition being tested plus one negative control condition had equal inputs of BFP-labeled resin. The DDM-treated EVs were then added to the appropriate resin tube and incubated for 1 h at 4 °C with end-over-end rotation, protected from ambient light. Each condition was then washed twice with buffer CIB for 5 min at 4C with end-over-end rotation, resuspended in 45 μL of buffer CIB, and stored on ice, protected from light. Each condition was sequentially pipetted with a cut pipette tip onto a glass microscope slide (Fisher) and gently covered with an 18 mm x 18 mm no. 1 glass cover slip (Matsunami) layered on carefully to minimize trapped air bubbles. Montaged images of the resin beads were collected using an Axio Observer TIRF microscope in epifluorescence mode (Zeiss) with a Prime 95B camera (Photometrics) running SlideBook v. 6.0.24 software (3i). Custom scripts were then used to identify resin beads in each image using DAPI (BFP) signal to both identify beads and normalize fluorescence signal in the FITC (BODIPY-cholesterol) or CY5 (AlexaFluor647-cholesterol) channels. Images of all resin particles were manually reviewed to ensure the exclusion of air bubbles, or other non-resin fluorescent artifacts from downstream analysis. Analysis scripts have been made publicly available at https://github.com/tribell4310/bead-assay.

### Immunopurification Assays

Co-immunopurification assays from EVs containing fluorescently labeled Prom1 or Pcdh21 were performed as described above for the chol-IP assay, using montaged fluorescence imaging for sensitive and quantitative measurements. Prom1-mScarlet was imaged using CY3 and Pcdh21-mNeonGreen using FITC filter sets.

### BS3 Crosslinking Assay

Bis(sulfosuccinimidyl)suberate (BS3) powder (Thermo Fisher) was dissolved in buffer immediately before use in assays. Briefly, 2 mg of BS3 powder was dissolved to a concentration of 25 mM in 25 mM HEPES-NaOH pH 7.8, 150 mM NaCl, 0.5 mM CaCl_2_. Two-fold serial dilutions were then prepared in the same buffer down to a lowest stock concentration of 0.8 mM. The assay was started by combining 10 μL of purified EVs with 2.5 μL of each dilution of BS3. As a negative control condition, 10 μL of purified EVs were combined with 2.5 μL of buffer. Reactions were incubated at ambient temperature for 30 min, then Tris-HCl pH 7.4 was added to each reaction at a final concentration of 50 mM and incubated for 15 min to quench unreacted BS3. Samples were analyzed by SDS-PAGE and immunoblotting.

### Equilibrium Sucrose Gradient Sedimentation Analysis

Linear sucrose gradients were prepared in Seton 7030 tubes using 5% and 30% (w/v) sucrose dissolved in PBS buffer using a Gradient Station IP (BioComp). Freshly prepared gradients were loaded into an SW-41 Ti swinging bucket rotor and 200 μL of the purified EVs were layered atop gradients immediately prior to centrifugation. Samples were centrifuged for 5 h, 22,000 rpm, 4 °C, then fractionated into 13 fractions of 930 μL using the Gradient Station IP. Gradient fractions were analyzed by SDS-PAGE and immunoblotting, then quantified as described above.

### Cholesterol depletion with methyl-beta cyclodextrin

Methyl beta-cyclodextrin (mBCD) (Sigma Aldrich) was dissolved in sterile-filtered PBS buffer to a final concentration of 20 mM, and a two-fold serial dilution series was prepared. For each condition (0 mM, 2.5 mM, 5 mM, and 10 mM mBCD), purified EVs were mixed 1:1 with the appropriate mBCD dilution and incubated for 2 h at 37 °C. After the reaction was complete, the EVs were immediately re-purified over a qEV2-35 nm column (Izon) as described above. Prom1 and Ttyh1 content was assessed with equal volumes of re-purified EVs by SDS-PAGE and immunoblotting. Cholesterol in the purified samples was quantified using Amplex Red reagent (Thermo Fisher) according to the manufacturer’s protocol, with fluorescence measurements taken using a SpectraMax M5 plate reader (Molecular Devices). EV morphology was characterized by preparing NS-TEM grids with mBCD-treated samples and analyzing the resulting images, as described above.

## Supplementary Material

Supplement 1

## Data Availability

Electron microscopy datasets and all biochemical data are deposited in a Zenodo repository at doi:10.5281/zenodo.10034616. Custom software packages are available at https://github.com/tribell4310.

## Significance Statement

Mammalian cells dynamically shape the plasma membrane to achieve specialized functions. Prominin-1 (Prom1) is a membrane protein that promotes bending and shaping of the membrane, notably in photoreceptors. Here, the authors find that membrane cholesterol regulates the membrane bending activity of Prom1 in extracellular vesicles. Membrane bending can be tuned by altering cholesterol levels and may be coupled to different Prom1 *N*-glycosylation states. A distant homolog of Prom1 called Ttyh1 is also shown to form extracellular vesicles and supports more membrane bending than Prom1 though it does not bind cholesterol. This work unites the Prom and Ttyh protein families into a single functional group and helps explain how membrane cholesterol functionalizes prominin-family proteins for different roles in different cell types.
