# Supplementary material for: Cholesterol regulates plasma membrane bending by prominin-family proteins
Source: bioRxiv. 2023 Nov 10:2023.11.08.566258. Preprint. [Version 2] doi: 10.1101/2023.11.08.566258 (PMC10659291; doi:10.1101/2023.11.08.566258)
Supplement: Supplementary file 1 [file NIHPP2023.11.08.566258v2-supplement-1.pdf]

bioRxiv preprint doi: https://doi.org/10.1101/2023.11.08.566258; this version posted November 10, 2023. The copyright holder for this preprint (which was not certified by peer review) is the author/funder, who has granted bioRxiv a license to display the preprint in perpetuity. It is made available under aCC-BY 4.0 International license.

Supporting Information

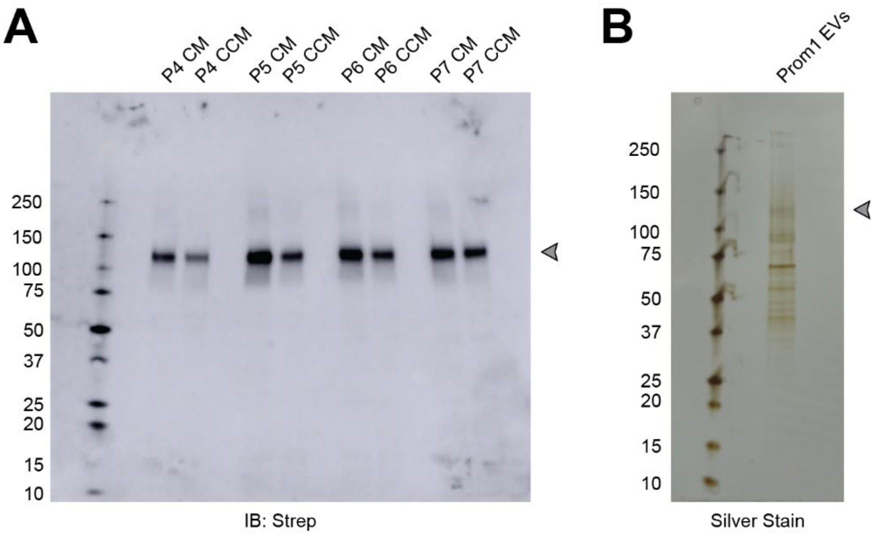

Figure S1. (A) Anti-Strep immunoblot of conditioned media (CM) or clarified conditioned media (CCM) from a stable polyclonal Expi293 cell line expressing lentiviral-transduced Prom1-Strep. Arrowhead indicates the expected position of glycosylated Prom1-Strep. P4, P5, P6, and P7 indicate the passage number of the suspension cell culture. **(B)** Total protein stain of purified Prom1-Strep EVs. Arrowhead indicates the expected position of glycosylated Prom1-Strep.

bioRxiv preprint doi: https://doi.org/10.1101/2023.11.08.566258; this version posted November 10, 2023. The copyright holder for this preprint (which was not certified by peer review) is the author/funder, who has granted bioRxiv a license to display the preprint in perpetuity. It is made available under aCC-BY 4.0 International license.

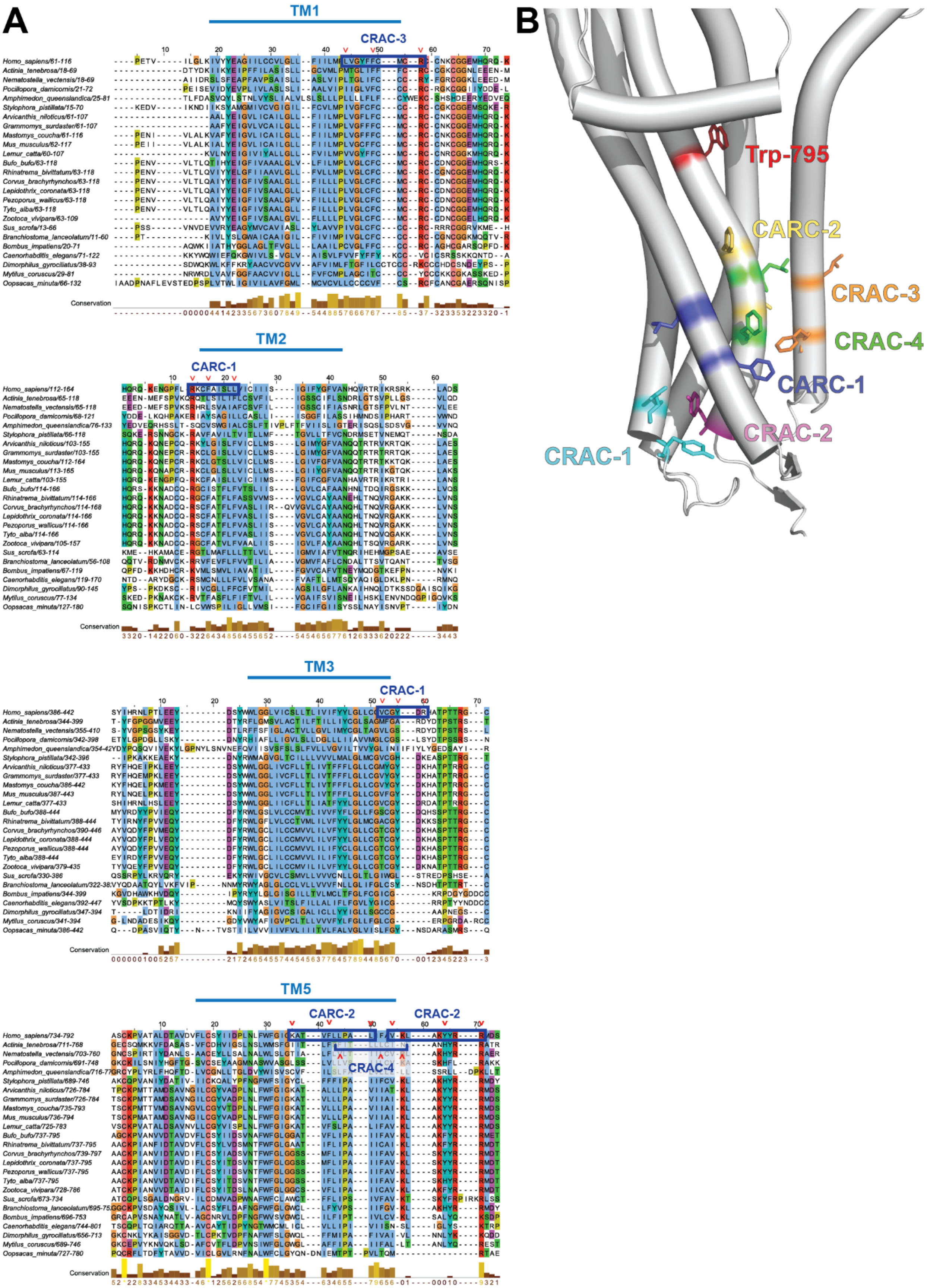

bioRxiv preprint doi: https://doi.org/10.1101/2023.11.08.566258; this version posted November 10, 2023. The copyright holder for this preprint (which was not certified by peer review) is the author/funder, who has granted bioRxiv a license to display the preprint in perpetuity. It is made available under aCC-BY 4.0 International license.

Figure S2. (A) Multiple sequence alignment of metazoan Prom1 focused on transmembrane domains 1, 2, 3, and 5, with human CRAC and CARC domains highlighted. Red carats indicate key charged, aromatic, or hydrophobic residues that define the CRAC and CARC domains. Alignment visualized using Jalview[64]. **(B)** Trp-795, CRAC-1, CRAC-2, CRAC-3, CRAC-4, CARC-1, and CARC-2 mutation sites superposed onto an AlphaFold2 model[46] of the transmembrane domain of Prom1.

bioRxiv preprint doi: https://doi.org/10.1101/2023.11.08.566258; this version posted November 10, 2023. The copyright holder for this preprint (which was not certified by peer review) is the author/funder, who has granted bioRxiv a license to display the preprint in perpetuity. It is made available under aCC-BY 4.0 International license.

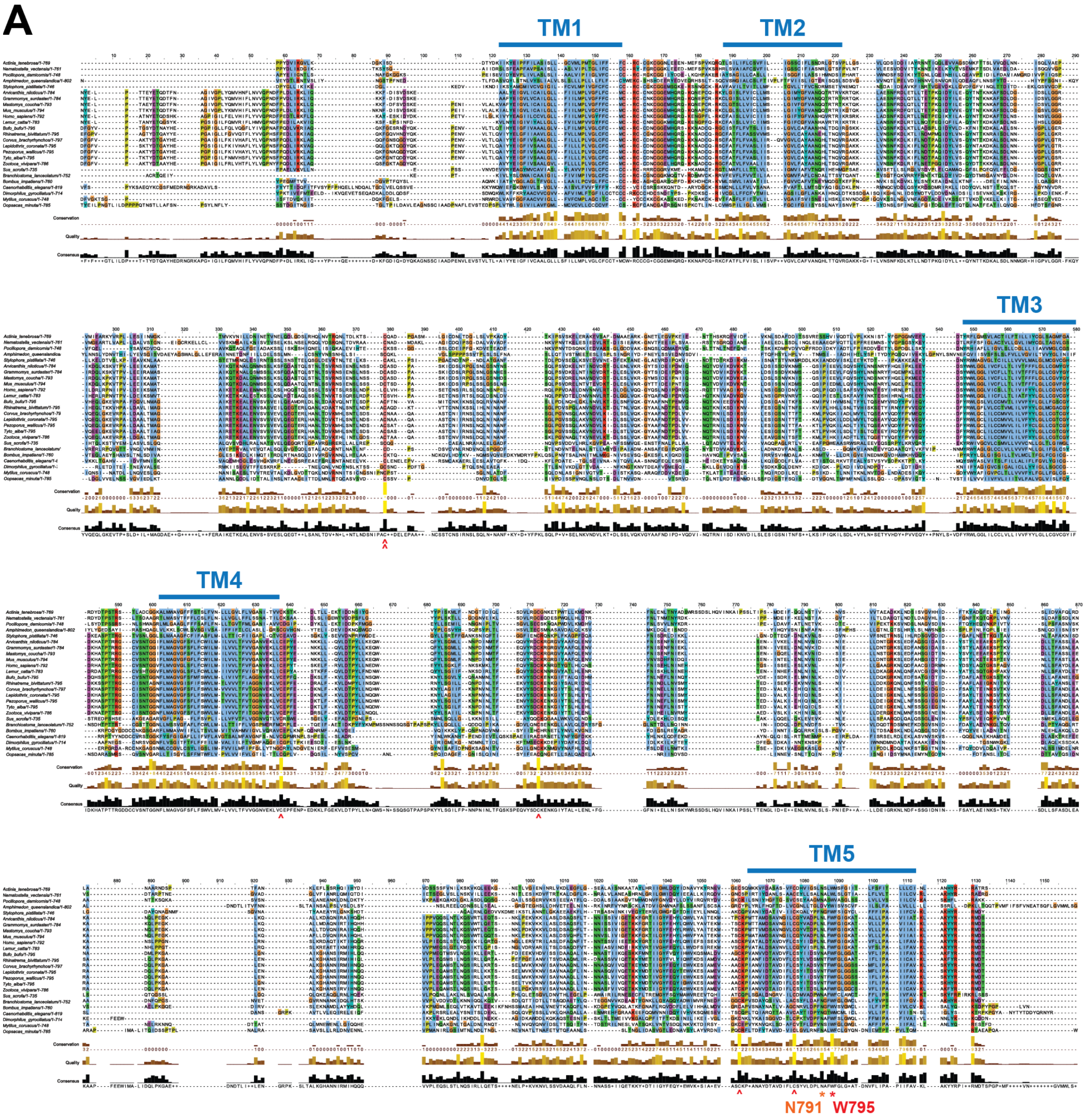

bioRxiv preprint doi: https://doi.org/10.1101/2023.11.08.566258; this version posted November 10, 2023. The copyright holder for this preprint (which was not certified by peer review) is the author/funder, who has granted bioRxiv a license to display the preprint in perpetuity. It is made available under aCC-BY 4.0 International license.

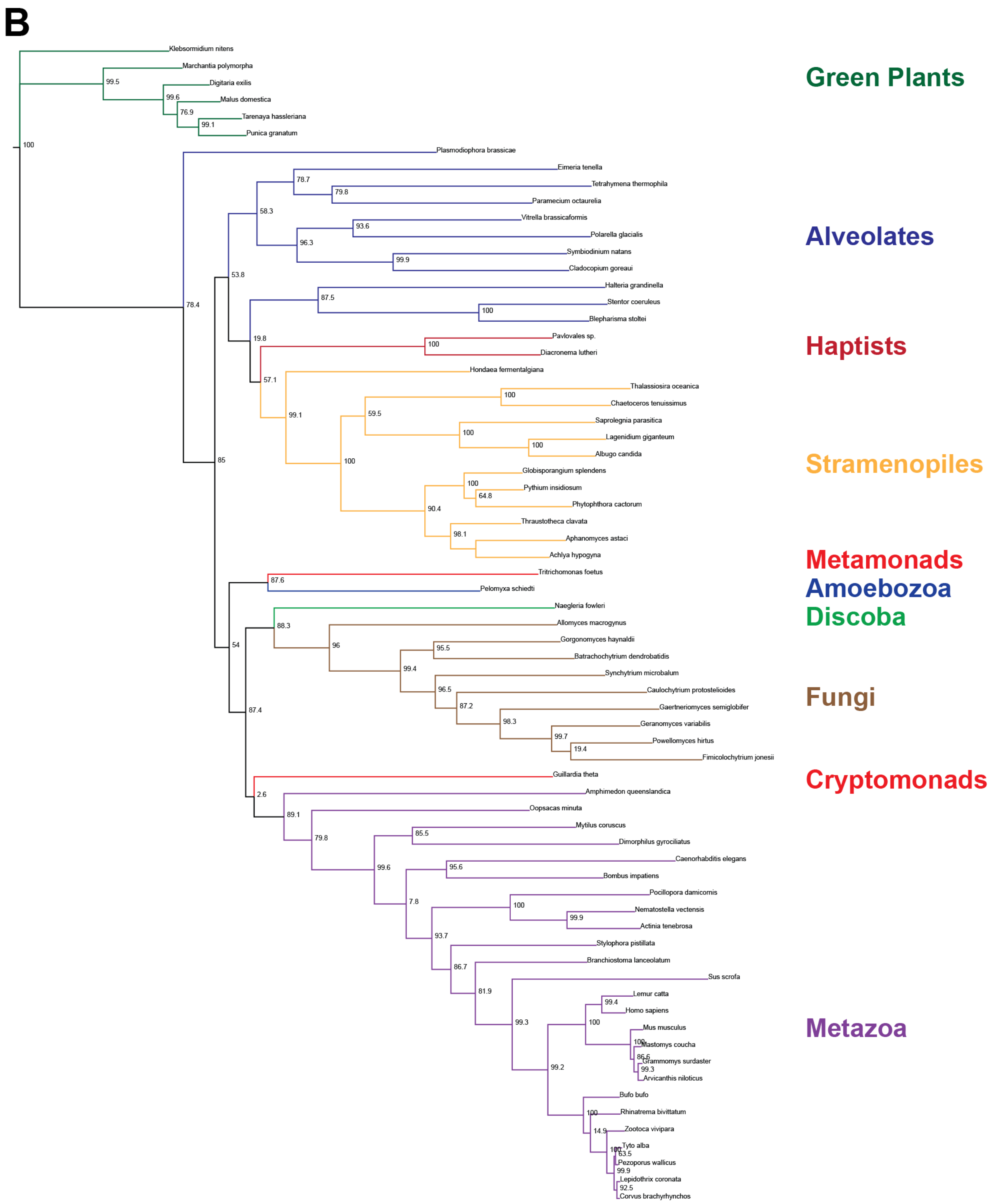

Figure S3. (A) Multiple-sequence alignment of prominin sequences from metazoa with the five transmembrane segments indicated. Red asterisk indicates perfectly conserved non-cysteine residues. Orange asterisk indicates less-than-perfectly conserved non-cysteine residues of interest. Red carat indicates cysteines predicted to form internal disulfides. Double red carat indicates cysteines that are not predicted to form internal disulfides. Alignment visualized using Jalview[64]. **(B)** Inferred phylogenetic

bioRxiv preprint doi: https://doi.org/10.1101/2023.11.08.566258; this version posted November 10, 2023. The copyright holder for this preprint (which was not certified by peer review) is the author/funder, who has granted bioRxiv a license to display the preprint in perpetuity. It is made available under aCC-BY 4.0 International license.

relationships between putative prominin homologs identified across eukaryotes. Node labels indicate ALRT branch supports[63]. Tree visualized using IcyTree[65].

bioRxiv preprint doi: https://doi.org/10.1101/2023.11.08.566258; this version posted November 10, 2023. The copyright holder for this preprint (which was not certified by peer review) is the author/funder, who has granted bioRxiv a license to display the preprint in perpetuity. It is made available under aCC-BY 4.0 International license.

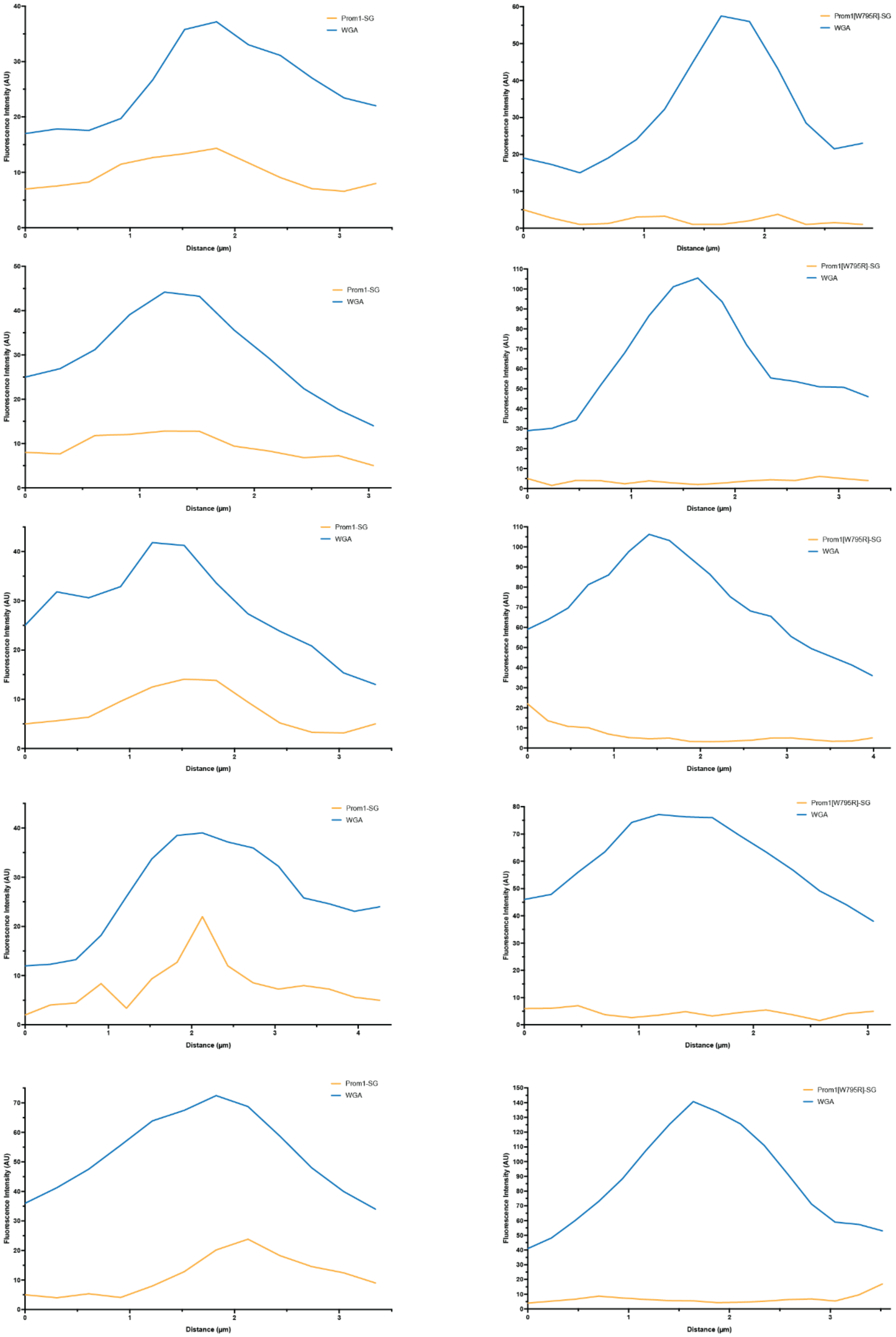

bioRxiv preprint doi: https://doi.org/10.1101/2023.11.08.566258; this version posted November 10, 2023. The copyright holder for this preprint (which was not certified by peer review) is the author/funder, who has granted bioRxiv a license to display the preprint in perpetuity. It is made available under aCC-BY 4.0 International license.

Figure S4. Line scan traces across cell junctions (n = 5) for plasma membrane (WGA) (blue) or Prom1-mStayGold fluorescence signal for WT (left panels) or W795R (right panels) Prom1 (yellow).

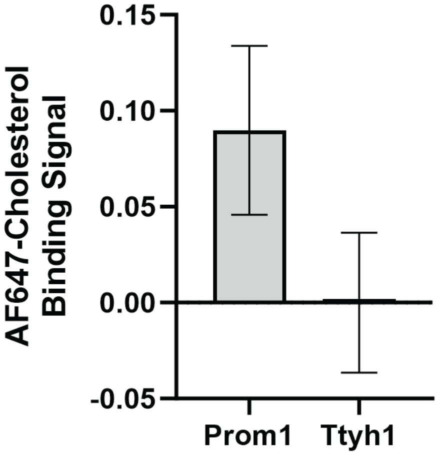

Figure S5. Cholesterol co-immunopurification (Chol-IP) measurement of AlexaFluor647-cholesterol binding in Prom1-Strep or Ttyh1-Strep EVs. Error bars indicate S.D. (n = 3).

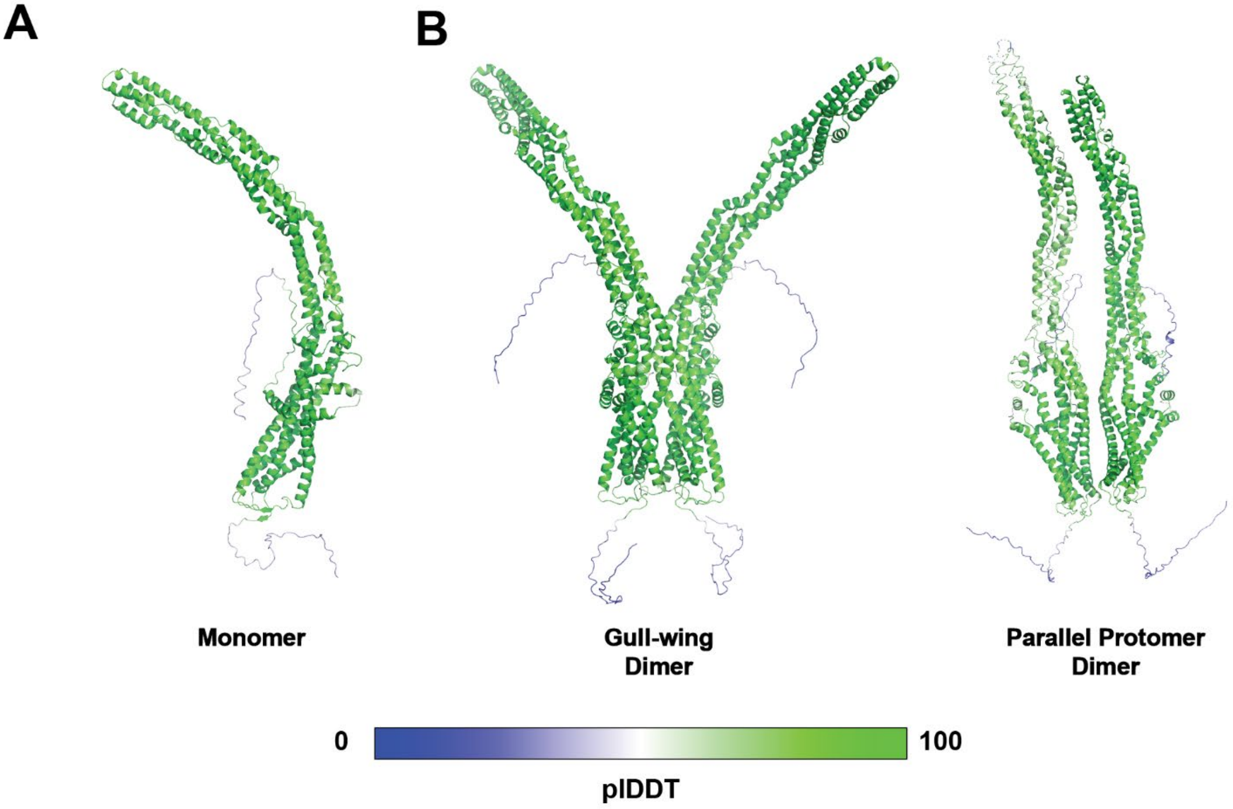

bioRxiv preprint doi: https://doi.org/10.1101/2023.11.08.566258; this version posted November 10, 2023. The copyright holder for this preprint (which was not certified by peer review) is the author/funder, who has granted bioRxiv a license to display the preprint in perpetuity. It is made available under aCC-BY 4.0 International license.

Figure S6. AlphaFold2[46] plDDT confidence scores mapped onto **(A)** Prom1 monomer model and **(B)** Prom1 dimer models.

Prom1 Mutant	Specific Mutations
Wild-type (WT)	- -
CRAC-1	V460A, Y463L
CRAC-2	Y819L
CRAC-3	L125A, F130L
CRAC-4	L806A, F811L
CARC-1	F158L, L162A, L163A
CARC-2	F804L, L806A, L809A
F62L	F62L
L161T	L161T
G454V	G454V
F505L	F505L
N791D	N791D
F794L	F794L
W795L	W795L
W795R	W795R
F796L	F796L

Table ST1. Prom1 mutants used in this study. All mutants are adapted from isoform S1 (NCBI accession NP_001139319.1).

Movie SM1. Live-cell confocal fluorescence microscopy of stable mStayGold-tagged WT Prom1 (Prom1-SG) (yellow) HeLa cells stained with wheat germ agglutinin (WGA) conjugated with Alexa Fluor 647 to label plasma membrane and intracellular vesicles (blue). Video consists of z-stack images acquired at 0.2 µm step size, assembled from basal to apical side.

Movie SM2. Live-cell confocal fluorescence microscopy of stable mStayGold-tagged W795R Prom1 (Prom1[W795R]-SG) (yellow) HeLa cells stained with wheat germ agglutinin (WGA) conjugated with Alexa Fluor 647 to label plasma membrane and intracellular vesicles (blue). Video consists of z-stack images acquired at 0.2 µm step size, assembled from basal to apical side.
